# Catalyst-loaded micro-encapsulated phase change material for thermal control of exothermic reaction

**DOI:** 10.1038/s41598-021-86117-1

**Published:** 2021-04-06

**Authors:** Tatsuya Takahashi, Hiroaki Koide, Hiroki Sakai, Daisuke Ajito, Ade Kurniawan, Yuji Kunisada, Takahiro Nomura

**Affiliations:** 1 Graduate School of Engineering, Hokkaido University, Kita 13 Nishi 8, Kita-ku, Sapporo, 060-8628 Japan; 2 Faculty of Engineering, Hokkaido University, Kita 13 Nishi 8, Kita-ku, Sapporo, 060-8628 Japan

**Keywords:** Catalysis, Chemical engineering, Energy, Materials chemistry

## Abstract

CO_2_ methanation is a promising technology to enable the use of CO_2_ as a resource. Thermal control of CO_2_ methanation, which is a highly active exothermic reaction, is important to avoid thermal runaway and subsequent degradation of the catalyst. Using the heat storage capacity of a phase change material (PCM) for thermal control of the reaction is a novel passive approach. In this study a novel structure was developed, wherein catalysts were directly loaded onto a micro-encapsulated PCM (MEPCM). The MEPCM was prepared in three steps consisting of a boehmite treatment, precipitation treatment, and heat oxidation treatment, and an impregnation process was adopted to prepare a Ni catalyst. The catalyst-loaded MEPCM did not show any breakage or deformation of the capsule or a decrease in the heat storage capacity after the impregnation treatment. MEPCM demonstrated a higher potential as an alternative catalyst support in CO_2_ methanation than the commercially available α-Al_2_O_3_ particle. In addition, the heat storage capacity of the catalyst-loaded MEPCM suppressed the temperature rise of the catalyst bed at a high heat absorption rate (2.5 MW m^−3^). In conclusion, the catalyst-loaded MEPCM is a high-speed, high-precision thermal control device because of its high-density energy storage and resolution of a spatial gap between the catalyst and cooling devices. This novel concept has the potential to overcome the technical challenges faced by efficiency enhancement of industrial chemical reactions.

## Introduction

Thermal control is essential for operating various systems at appropriate temperatures and preventing system failures. Thermal runaway is one of the problems caused by an inability to properly control the reaction temperature. It is a positive loop state wherein the heat of an exothermic reaction causes the catalyst temperature to rise, which in turn promotes the reaction and further increases the catalyst temperature. This phenomenon occurs in chemical reactors and batteries and eventually results in the loss of control over the system and possible explosion^1–3^. Thermal runaway in a heterogeneous catalytic reaction is also an economical problem because it reduces product selectivity, changes product distribution, and reduces the activity and life span of the catalyst^2^. Industrial fixed-bed reactors are operated at relatively high temperatures for the efficient conversion of reactants to products^1^. In this case, the temperature of the hot spot in the catalyst layer is close to the unstable range, and a slight disturbance in operating conditions can cause thermal runaway.

As one of the promising thermal control technologies, latent heat storage (LHS), which exploits the latent heat at the solid–liquid phase transformation of a phase change material (PCM), has attracted attention for its variety of positive characteristics, such as the release and absorption of a large amount of heat within a small temperature range, high repeatability, and heat transfer at a constant temperature^4–7^. Therefore, LHS behaves as an isothermal heat sink when the operation temperature matches the melting temperature of the PCM and precise temperature control within a narrow range is expected. Furthermore, passive thermal control, including LHS, is used more often than active thermal control owing to its higher operational reliability and lower cost^8,9^. However, the available temperature range of LHS depends mainly on the type of PCM used^10,11^. Typically, molten salts or alloys are used in the high temperature range that favors a chemical reaction^4,12,13^. Alloys are more promising than molten salts because of their high thermal conductivity, low volume expansion, and minimal supercooling^12,13^. Some alloy PCMs have been reported recently. Blanco-Rodríguez et al. reported a Mg–Zn alloy, and Risueño et al. investigated Mg–Zn–Al alloys^14,15^. In several of these studies, the leakage of molten PCM is a problem, and encapsulation of the PCM is essential to prevent it^16^.

Our research group previously developed a micro-encapsulated PCM (MEPCM) consisting of an Al–Si alloy core and an α-Al_2_O_3_ shell^17–19^. The MEPCM exhibited a high melting point above 500 °C, high heat storage density of 180 J g^−1^ or higher, and durability over 3000 cycles. Furthermore, microscale encapsulation provided beneficial properties for thermal control: miniaturization of the PCM enabled rapid melting of the internal alloy^2^, and expansion of the heat exchange area provided rapid thermal response^20^.

Several thermal control technologies utilizing LHS have been reported. Zhang et al. used In@SiO_2_ capsules as a thermally functional additive to suppress the generation of local hot spots^2^. Odunsi et al. investigated a simulation of the Fischer–Tropsch reaction using PCM, wherein the reaction bed temperature was maintained in the appropriate range^1^. LHS is also used to maintain the system temperature and to balance the heat demand and supply. Gokon et al. proposed a PCM-solar reactor to suppress the temperature change of a catalyst bed due to solar radiation fluctuation^21^; high catalyst performance was maintained for 30 min during the heat-discharging mode. Pattison et al. proposed autothermal microchannel reactors in which the heat of the exothermic reaction was used in the endothermic reaction^22^. They confined the PCM layer between each reactor to suppress the thermal imbalance of the two reactions. On the other hand, when PCM is used as an additive in the catalyst layer or as a reactor jacket, the spatial gap between the heat source and PCM inhibits efficient heat conduction. Hence, the contact structure between the PCM surface and heat source is important for thermal control^23^. Li et al. demonstrated the heat management of a chemical loop combustion in contact with Al@Al_2_O_3_ PCM microcapsules^23^. However, there are few studies on thermal management components where a heat source was loaded onto the PCM capsule.

**Figure 1:**
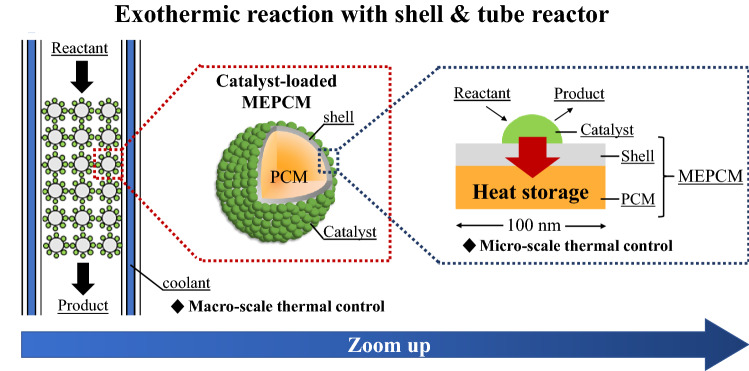
Schematic diagram of the catalyst-loaded MEPCM and the thermal control system.

Therefore, a structure in which the catalyst acts as a heat source for the exothermic reaction when loaded onto the surface of an MEPCM is a novel and promising heat control material. Ceramic materials, such as SiO_2_, Al_2_O_3_, and TiO_2_, are often used as catalyst supports due to their high heat resistance and chemical stability^24^. Since the MEPCM shell consists of α-Al_2_O_3_, it can be used as a catalyst support. Figure 1 shows a schematic diagram of the catalyst-loaded MEPCM and the thermal control system. The MEPCM, which is covered with fine particles of the catalyst, removes the heat generated on the catalyst surface during exothermic reactions at the nanoscale because of its heat storage capacity. Catalyst-loaded MEPCMs are used in combination with typical cooling devices (e.g., shell and tube reactor^25^). These units cool the entire catalyst layer and prevent the PCM from losing its thermal storage characteristics due to a complete phase transformation. Thus, the catalyst-loaded MEPCM is expected to control the temperature of the entire catalyst layer at the nanoscale.

CO_2_ methanation (${\rm{C}}{{\rm{O}}_{\rm{2}}}{\rm{ + 4}}{{\rm{H}}_{\rm{2}}}{\rm{ = C}}{{\rm{H}}_{\rm{4}}}{\rm{ + 2}}{{\rm{H}}_{\rm{2}}}{\rm{O(g),\;}} \delta {\rm{H}}_{{\rm{298K}}}^{\rm{0}}{\rm{ = }} - {\rm{164\;kJ\;mo}}{{\rm{l}}^{ - {\rm{1}}}})$ is considered as an important application of the catalyst-loaded MEPCM because of its demand for high thermal control^26^. It presents an alternative to conventional natural gas production by converting CO_2_ to CH_4_, which is effective for reducing CO_2_ emissions^27^. Additionally, CH_4_ is superior to H_2_ in terms of capital expenditure as transportation of CH_4_ can be achieved by using existing natural gas pipelines^27,28^. Based on the above, a power-to-gas system employing CO_2_ methanation has been previously proposed^25,29,30^. CO_2_ methanation requires catalysts (Ni, Rh, Ru, etc.), and Ni, which is the most widely used, shows high catalytic activity from 300 to 400 °C^28,31^. However, thermal runaway is a concern due to the highly exothermic reaction^32–34^. The use of a Ni-loaded MEPCM (Ni/MEPCM) instead of a conventional catalyst may suppress a significant rise in temperature.

The purpose of this study was to fabricate Ni/MEPCM and investigate its catalytic activity for CO_2_ methanation and its thermal control characteristics during an exothermic reaction. Ni/MEPCM was fabricated by the conventional impregnation method because the MEPCM surface was assumed to behave in a manner similar to that of the surface of α-Al_2_O_3_ powder. A CO_2_ methanation reaction was conducted to measure the catalytic performance. Based on the results, CO_2_ conversion (%) and CH_4_ selectivity (%) were estimated.

## Methods

### Preparation of MEPCM

MEPCM was prepared from Al-Si microspheres (25 mass% Si; Diameter: < 38 $\mathrm{\mu m}$, Purity: 99.0%, Hikari Material Industry Co. Ltd.). The melting point of the composition is approximately 577 °C. The encapsulation of the microspheres was performed in three steps consisting of a boehmite treatment, precipitation treatment, and heat oxidation treatment, as proposed in a previous study^19^. First, 300 mL of distilled water containing 1.0 g of Al(OH)_3_ (Purity: 99.99%, Kojundo Chemical Lab. Co., Ltd.) was boiled with a hot stirrer. After adjusting the solution to a pH of 8.0 by adding a 1 M solution of NH_3_, 10 g of the Al-Si microspheres were added to the water. The mixture was continuously stirred for 3 h to form AlOOH shells on the surfaces of the microspheres by the hydration reaction. Second, the solutions were cooled to 75 °C and maintained at this temperature for 16 h. Subsequently, the precursor samples were filtered and dried at 105 °C overnight. Finally, 10 g of the precursor samples were heated from room temperature to 1150 °C at a rate of 10 °C min^−1^ and maintained there for 6 h under an O_2_ gas flow (Purity: 99.5%, rate: 2.0 L min^−1^) to form the α-Al_2_O_3_ shells.

### Preparation of Ni/MEPCM

Ni loading onto the MEPCM was carried out by the impregnation method, here, the raw materials were adjusted so that the amount of nickel supported in the final product was 10%. An aqueous solution containing 5.0 mL of distilled water and 0.50 g of Ni(NO_3_)_2_·6H_2_O (Purity: 99.9%, Kojundo Chemical Lab. Co., Ltd.) was added to 0.90 g of the as-prepared MEPCM. The mixture was crystallized at 70 °C by a rotary evaporator (Shibata Scientific Technology Ltd, SRE-M3). The impregnated sample was dried at 105 °C for 24 h, then calcined at 500 °C for 4 h to prepare the Ni/MEPCM. Ni/α-Al_2_O_3_ was also prepared for comparison by adding 0.90 g of α-Al_2_O_3_ (Average Diameter = 28 μm, Purity: 99.9%, Showa Denko Co., Ltd.) into another aqueous solution of distilled water and Ni(NO_3_)_2_·6H_2_O, as before, and the mixture was crystallized at 70 °C. The impregnated sample was then dried at 105 °C for 24 h and calcined at 500 °C for 4 h to prepare the Ni/α-Al_2_O_3_ reference.

### Sample characterization

The phase compositions of the samples were characterized using X-ray powder diffraction (XRD, Rigaku, Miniflex600, Cu Kα). The surface chemical state was analyzed using X-ray photoelectron spectroscopy (XPS, JEOL, JPS-9200). XPS spectra were acquired using an Al Kα X-ray source (1486.6 eV). The calibration was carried out by referring to Au 4f_7/2_ (84.00 eV), Cu 2p_3/2_ (932.63 eV), and Ag 3d_5/2_ (368.28 eV). The morphology was observed by scanning electron microscopy-energy dispersive spectroscopy (SEM–EDS, JEOL, JSM-7400F). Brunauer–Emmett–Teller (BET) specific surface areas of the samples were analyzed based on the N_2_ adsorption/desorption techniques using a gas adsorption analyzer (Yuasa Ionics, Autosorb 6AG). The density of the samples was measured using a gas pycnometer (Quantachrome instruments, Ultrapycnometer1000). Additionally, the phase transition temperature and thermal storage ability were measured using a differential scanning calorimetry analyzer (DSC, Mettler-Toledo, TGA/DSC3 +). The MEPCM sample was placed in an Al_2_O_3_ crucible and heated from room temperature to 600 °C at a rate of 5.0 °C min^−1^ and maintained at this temperature for 5 min under an Ar gas flow (Purity 99.5%, rate 50 mL min^−1^).

### CO_2_ methanation

**Figure 2:**
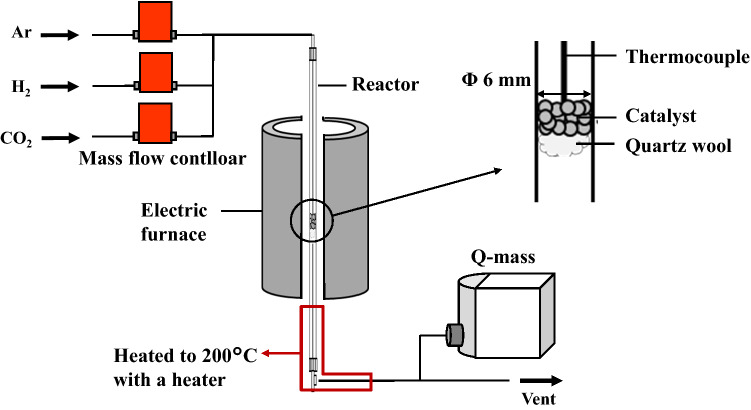
Schematic diagram of the vertical fixed bed tubular reactor.

CO_2_ methanation was performed in a vertical fixed bed tubular reactor (inner diameter: 6.0 mm) at atmospheric pressure to evaluate the catalytic activity tests. Figure 2 shows a schematic diagram of the reactor. One-tenth of a gram of the samples were loaded into the reactor and fixed with quartz wool. The samples were reduced for 1 h at 500 °C under H_2_ and Ar gas flow (${\text{F}}_{\text{in, }{\text{H}}_{2}}$= 40 mL min^−1^ and ${\text{F}}_{\text{in, Ar}}$= 110 mL min^−1^, where ${\text{F}}_{\text{in, }{\text{x}}}$ represents the flow rate of the inlet gas). After cooling to the reaction temperature in Ar gas flow, the feed gas (${\text{F}}_{\text{in, }{\text{CO}}_{2}}$= 10 mL min^−1^, ${\text{F}}_{\text{in, }{\text{H}}_{2}}$= 40 mL min^−1^, and ${\text{F}}_{\text{in, Ar}}$= 110 mL min^−1^) was introduced into the reactor for 1 h at 400 °C. The concentrations of the outlet gas was analyzed using a Quadrupole mass spectrometer (Q-mass, Pfeiffer Vacuum, Thermostar GSD301). CO_2_ conversion and CH_4_ selectivity were calculated using Eqs. (1) and (2):

1 $${\rm{C}}{{\rm{O}}_{\rm{2}}}{\rm{conversion\;(\% ) = }}\frac{{\left( {{{\rm{F}}_{{\rm{in,\;C}}{{\rm{O}}_{\rm{2}}}}} - {{\rm{F}}_{{\rm{out,\;C}}{{\rm{O}}_{\rm{2}}}}}} \right){\rm{ \times 100}}}}{{{{\rm{F}}_{{\rm{in,\;C}}{{\rm{O}}_{\rm{2}}}}}}}$$

2 $${\rm{C}}{{\rm{H}}_{\rm{4}}}{\rm{\;selectivity}}\left( {\rm{\% }} \right){\rm{ = }}\frac{{{{\rm{F}}_{{\rm{out,\;C}}{{\rm{H}}_{\rm{4}}}}}{\rm{ \times 100}}}}{{\left( {{{\rm{F}}_{{\rm{out,\;C}}{{\rm{H}}_{\rm{4}}}}}{\rm{ + }}{{\rm{F}}_{{\rm{out,\;CO}}}}} \right)}},$$

where ${\text{F}}_{\text{out, }{\text{x}}}$ represents the flow rate of the outlet gas.

### Measurement of thermal control performance

The CO_2_ methanation reaction was also used as a test to evaluate the thermal control characteristics of the Ni/MEPCM material. The reactions were carried out with Ni/MEPCM under two reaction conditions, and the temperature of the sample bed was measured in each case. The difference between the two conditions determined whether the internal PCM was a solid or liquid (i.e., whether the samples had a heat storage capacity or not). The temperature control performance of the PCM was evaluated from the difference between these temperatures.

**Figure 3:**
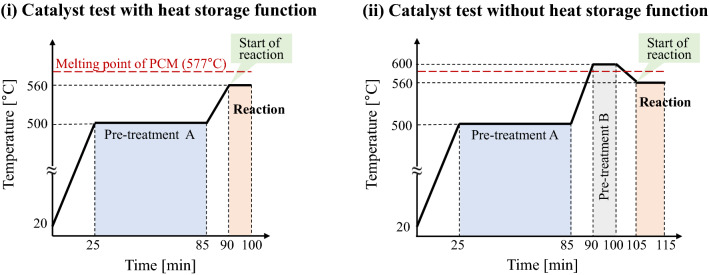
Temperature programs for measuring thermal control performance during the CO_2_ methanation reaction of the Ni/MEPCM with/without a heat storage function. In the condition (i), internal PCM is solid and has the latent heat storage function when the catalytic reaction starts. In the condition (ii), internal PCM is liquid and does not have the latent heat storage function when reaction starts. Here, Pre-treatment (**A**) is reduction of NiO of the as prepared Ni/MEPCM under H_2_ and Ar flow at 500 °C. Pre-treatment (**B**) is for loosing heat storage function of the Ni/MEPCM by maintain it at 600 °C for 10 min in Ar flow. CO_2_ methanation reaction was started at 560 °C after Pre-treatment (**A**) or (**B**).

The reaction was carried out in the same reactor as in previous section, and the thermocouple was inserted into the middle of the sample bed to measure the temperature of the sample. Two-tenths of a gram of the Ni/MEPCM were loaded into the reactor such that the tip of the thermocouple was sufficiently covered by the samples. Figure 3 shows temperature programs for measuring thermal control performance during the CO_2_ methanation reaction of the Ni/MEPCM with/without a heat storage function. In condition (i), when the temperature of the sample layer exceeded the melting point of the PCM by the reaction heat, the phase transformation of PCM occurred, and heat storage began. In condition (ii), the PCM melted before the reaction started, and the liquid state was maintained even at the reaction temperature for supercooling. Therefore, heat storage did not occur during the reaction. H_2_ and Ar gas (${\text{F}}_{\text{in, }{ \, {\text{H}}}_{2}}$= 40 mL min^−1^ and ${\text{F}}_{\text{in, Ar}}$= 110 mL min^−1^) flowed during the reduction, and the feed gas (${\text{F}}_{\text{in, }{\text{ CO}}_{2}}$= 15 mL min^−1^, ${\text{F}}_{\text{in, }{ \, {\text{H}}}_{2}}$= 60 mL min^−1^, and ${\text{F}}_{\text{in, Ar}}$= 50 mL min^−1^) was introduced during the reaction.

## Results and discussion

### Catalyst characterization

#### Phase composition of samples

**Figure 4:**
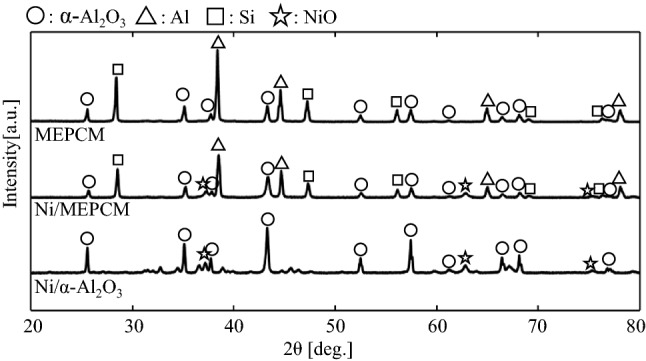
X-ray diffraction (XRD) patterns of the MEPCM, Ni/MEPCM, and Ni/α-Al_2_O_3_.

Figure 4 shows the XRD patterns of the MEPCM, Ni/MEPCM, and Ni/α-Al_2_O_3_ samples. Peaks of Al, α-Al_2_O_3_, and Si were detected in the results of the MEPCM and Ni/MEPCM, and peaks of NiO was observed in the Ni/MEPCM. Peaks corresponding to α-Al_2_O_3_ and NiO were detected in the Ni/α-Al_2_O_3_. Furthermore, the weight ratios of NiO in each sample were calculated using reference intensity ratio (RIR) analyses. The weight ratios of NiO were 7.9% for Ni/MEPCM and 8.5% for Ni/α-Al_2_O_3_.

The XRD peaks of Al and Si were attributed to the core alloy in the MEPCM; meanwhile, the α-Al_2_O_3_ peak was attributed to its shell. Stronger peaks of α-Al_2_O_3_ were observed in the Ni/α-Al_2_O_3_ than in the other samples. This is due to the fact that only the shell of the MEPCM was composed of α-Al_2_O_3_ as opposed to the entire structure of Ni/α-Al_2_O_3_. Since the peaks of NiO and α-Al_2_O_3_ were similar in all samples, the prepared samples were expected to have similar catalytic properties. The weight ratios of the Ni loaded on the two samples were 6.2% for Ni/MEPCM and 6.7% for Ni/α-Al_2_O_3_, based on the weight ratios of NiO calculated by the RIR analysis. Although the amount of loaded Ni was smaller than that of the input raw material, the amount of loaded Ni on the Ni/MEPCM and Ni/ α-Al_2_O_3_ was similar in extent.

#### Morphology of samples

**Figure 5:**
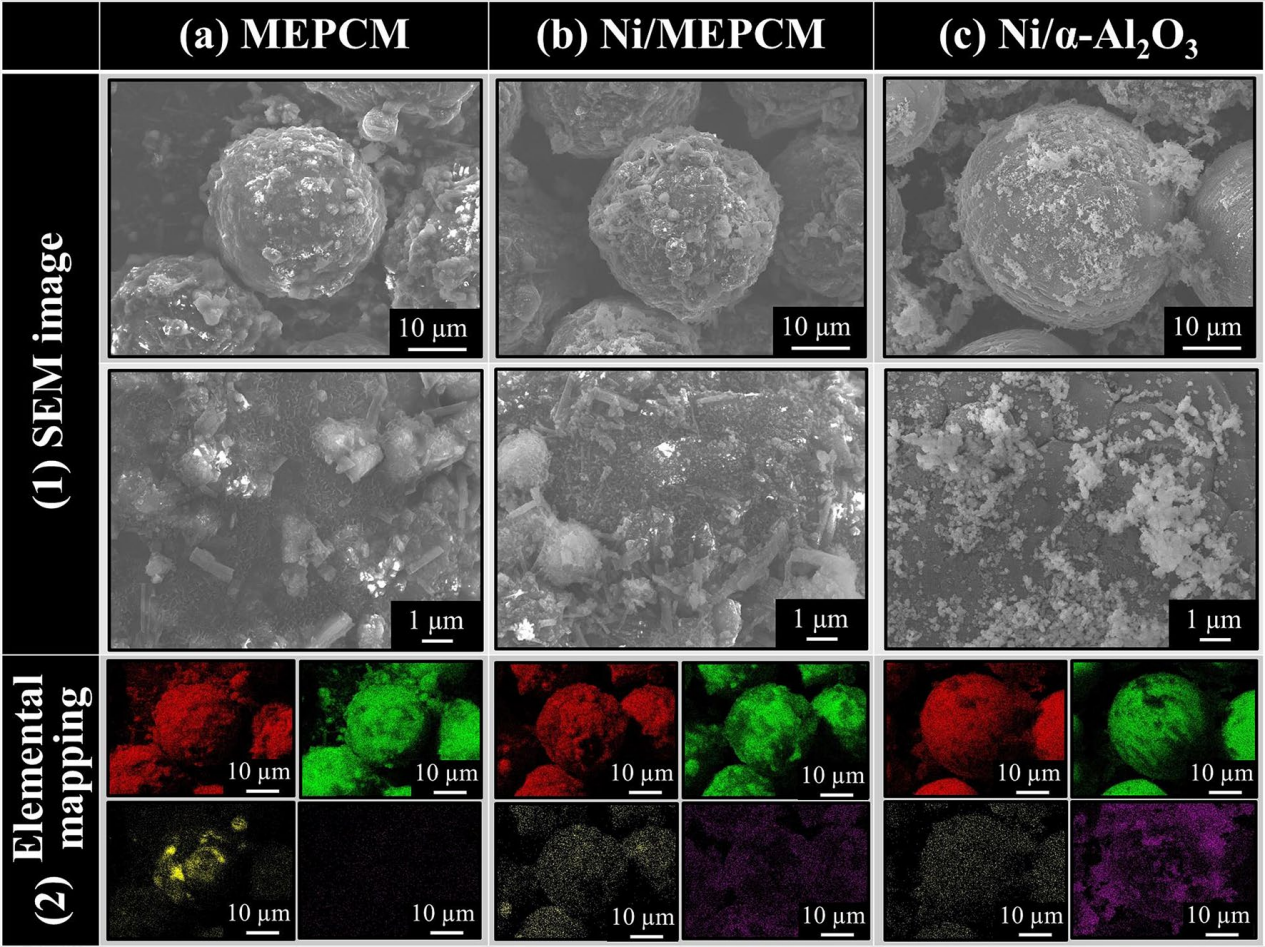
Scanning electron microscopy (SEM) image and the Energy dispersive spectroscopy (EDS) elemental mapping of the (**a**) MEPCM, (**b**) Ni/MEPCM, and (**c**) Ni/α-Al_2_O_3_. In the elemental mapping, the red area represents O, the green area represents Al, the yellow area represents Si and the purple area represents Ni.

Figure 5 shows the SEM images and elemental mapping results of the MEPCM, Ni/MEPCM, and Ni/α-Al_2_O_3_ samples. The entire surface of the MEPCM was covered with a needle-like structure, and cuboid crystals had formed on the structure. The same structure along with fine particles of approximately 40 nm in diameter were present in the Ni/MEPCM. A smooth surface was detected in the Ni/α-Al_2_O_3_, and fine particles of approximately 100 nm in diameter were present on its surface. In the elemental mapping, the MEPCM shell was composed of Al, O, and a small amount of Si, while Ni was present on the MEPCM shell after Ni impregnation in the Ni/MEPCM. In contrast, Al, O, and Ni were strongly detected in the Ni/α-Al_2_O_3_.

The needle-like structure and cuboid crystals were composed of Al and O, and the XRD patterns reveal α-Al_2_O_3_ peaks. Thus, the MEPCM shells had formed only from α-Al_2_O_3_. The characteristic needle-like structures corresponded to AlOOH formed during the boehmite treatment^17^. The cuboid crystals represented the deposited or adhered Al(OH)_3_ particles formed during the precipitation treatment^19^. These two types of structures created a coarse configuration. The small amount of Si that was detected implied that most of the Si was present only in the MEPCM core. The fine particles were present only in the Ni-impregnated sample; thus, they were considered to be NiO from the elemental mapping and XRD patterns. Ni was supported on the MEPCM without aggregation since these particles were distributed. As the core/shell structure was neither broken nor deformed, and there was no elemental mapping of Si outside the capsules, no apparent core leakage occurred during the impregnation process. On the other hand, in Ni/α-Al_2_O_3_, the particles recognized as NiO were more aggregated than those in the Ni/MEPCM, and Si was determined as a trace impurity in the production.

#### Surface chemical state

**Figure 6:**
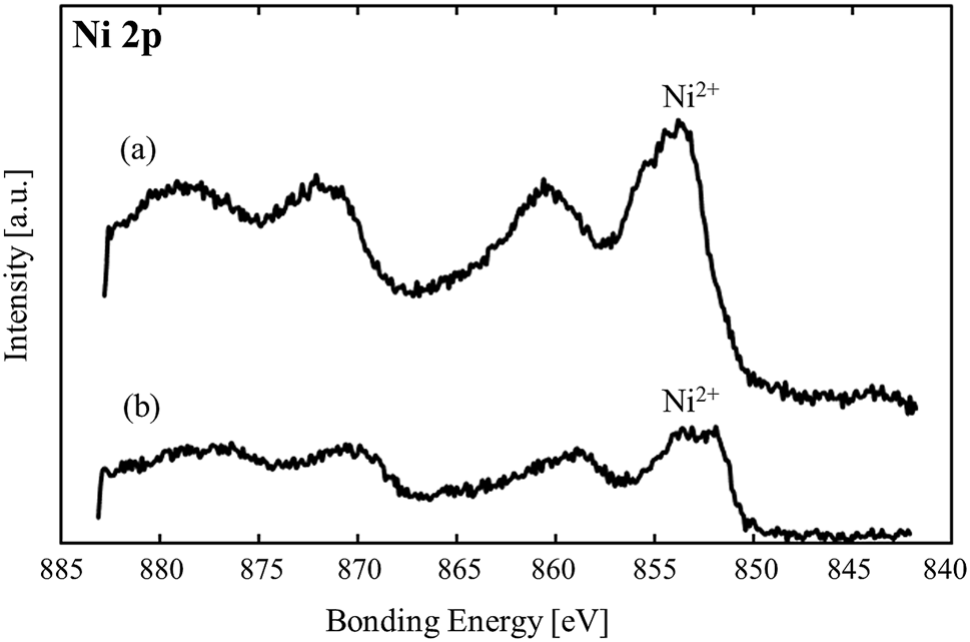
Ni 2p region of the XPS spectra for the (**a**) Ni/MEPCM and (**b**) Ni/α-Al_2_O_3_.

Figure 6 shows the Ni 2p region of the XPS spectra for the Ni/MEPCM and Ni/α-Al_2_O_3_. The peak shift associated with the charging of the sample was already uniformly corrected on the basis of the C 1 s (285 eV). In both samples, strong peaks were observed in a binding range of 853.5–854 eV.

In both samples, Ni particles on the surface were mainly in the form of Ni^2+^ because the strongest peak was observed at 853.5–854 eV. Furthermore, the loss peak (860 eV) was derived from NiO. The complex shape of the catalyst-loaded MEPCM may have different surface charges depending on the site. Therefore, the difference in the peak positions between the two samples is considered a result of the charging of the samples and could not be corrected. During the measurement, the core alloy was solid as XPS analysis was conducted at a low temperature. In contrast, the core alloy melts by the heat of the exothermic reaction. The molten Al–Si alloy is suspected to affect the loaded Ni due to the thin shell of the MEPCM. Therefore, it is possible that the chemical state of the Ni particles changed during the exothermic reaction, and it is necessary to examine this in future studies.

#### BET specific surface area of samples

Table 1 shows the BET specific surface areas and densities of the MEPCM, Ni/MEPCM, and Ni/α-Al_2_O_3_. A difference in the interior composition of the Ni/MEPCM and Ni/α-Al_2_O_3_ caused a difference in mass even at equal volumes. Therefore, these samples should be evaluated by the BET specific surface area per volume, which was converted from the specific surface area per mass using the density of each sample. The Ni/MEPCM had the largest BET specific surface area at 38.2 × 10^6^ m^2^ m^−3^.

**Table 1:** BET specific surface areas per mass or volume, and densities of the MEPCM, Ni/MEPCM, and Ni/α-Al_2_O_3_.

	MEPCM	Ni/MEPCM	Ni/α-Al_2_O_3_
BET surface area (m^2^ g^−1^)	10.19	11.28	2.75
Density (g m^−3^)	2.69 × 10^6^	3.39 × 10^6^	4.11 × 10^6^
BET surface area (m^2^ m^−3^)	27.4 × 10^6^	38.2 × 10^6^	11.3 × 10^6^

The Ni/MEPCM showed a slightly larger surface area than that of the pure MEPCM since the crystals were formed on the surface of the MEPCM. At 11.3 × 10^6^ m^2^ m^−3^, the surface area of the Ni/α-Al_2_O_3_ was smaller than that of the MEPCM owing to the smoother surface morphology of the α-Al_2_O_3_ particles. Generally, α-Al_2_O_3_ has a small specific surface area due to the formation process of α-Al_2_O_3_, whereby the high-temperature heat treatment facilitates the rearrangement of the Al_2_O_3_ particles and reduces the surface area^35^. In contrast, the MEPCM had a higher specific surface area than that of ordinary α-Al_2_O_3_ powder because of its rough structure. Therefore, MEPCM is superior to α-Al_2_O_3_ powder as a catalyst support in terms of active-catalyst dispersion.

### Thermal properties

**Figure 7:**
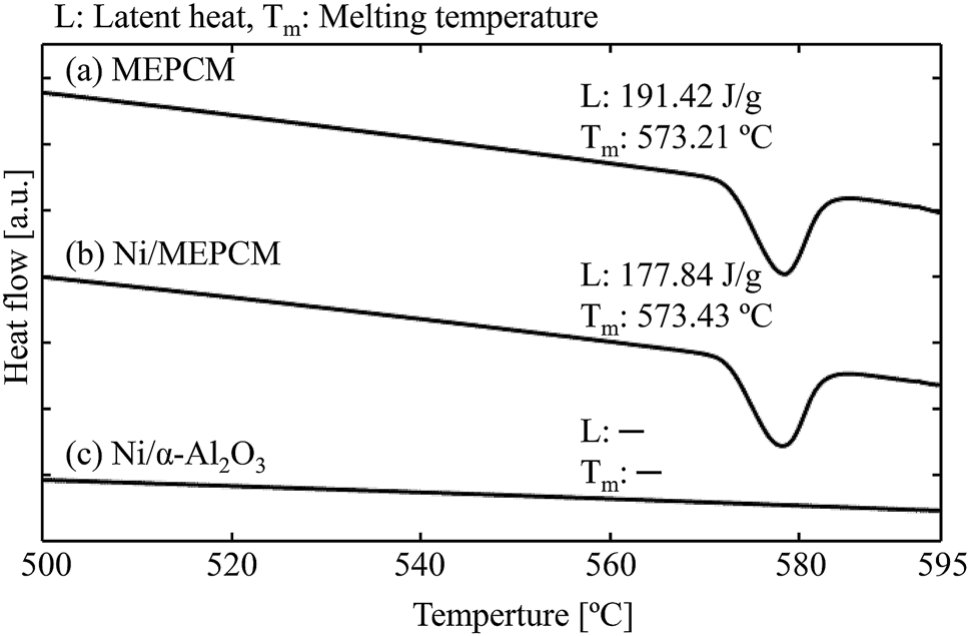
Heating DSC curves of the MEPCM, Ni/MEPCM, and Ni/ α-Al_2_O_3_.

Figure 7 shows the DSC curves during heating of the MEPCM, Ni/MEPCM, and Ni/α-Al_2_O_3_ samples. An endothermic peak at approximately 573 °C is near the eutectic temperature of Al-Si alloy in both the MEPCM and Ni/MEPCM. Also, the LHS capacities of the MEPCM and Ni/MEPCM were different at 191.42 and 177.84 J g^−1^, respectively. No endothermic peak was observed in the Ni/α-Al_2_O_3_.

Since the onset temperature of the endothermic peak was close to the eutectic temperature (577 °C) of the Al-Si alloy, the endothermic peak arose from the phase change of the core alloy. Because Ni impregnated on the MEPCM as NiO particles (at 10 mass% Ni), the LHS capacity of the Ni/MEPCM was 203.77 J g^−1^ if NiO was neglected. This value was not less than that of the MEPCM before Ni impregnation. Therefore, Ni does not affect the heat storage capacity of the MEPCM.

### CO_2_ methanation

**Figure 8:**
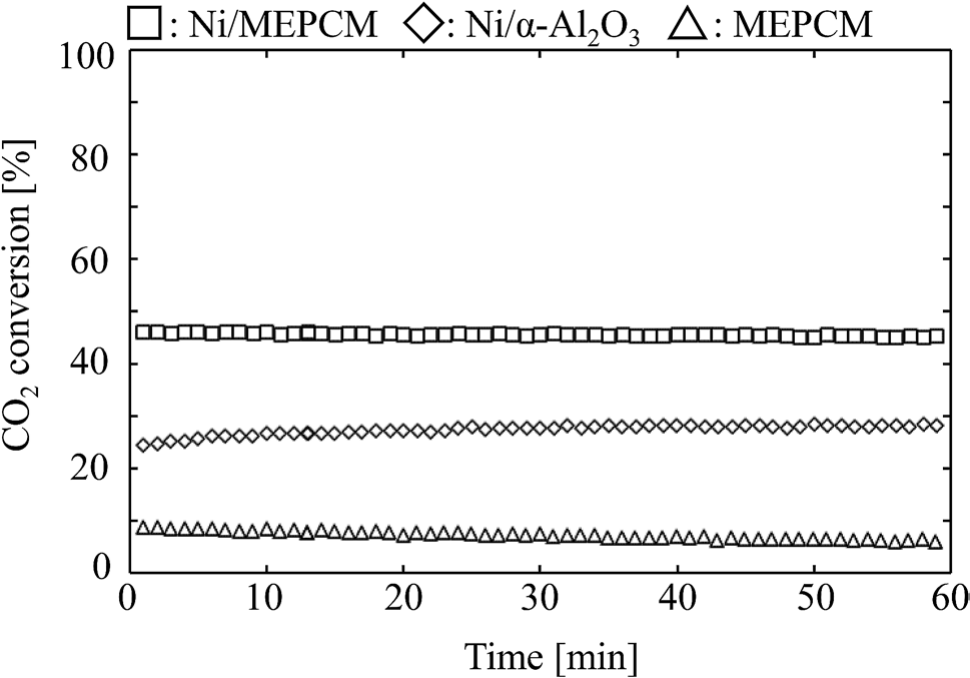
CO_2_ conversion of the Ni/MEPCM, Ni/α-Al_2_O_3_, and MEPCM. Reaction condition: ${F}_{in, {CO}_{2}}$= 10 ml min^−1^, ${F}_{in, {H}_{2}}$= 40 ml min^−1^, ${F}_{in, Ar}$= 110 ml min^−1^, Reaction temperature: 400 °C.

The catalytic activity of each sample was evaluated by analyzing the outlet gas with a Q-mass during CO_2_ methanation. Figure 8 shows the CO_2_ conversion of each catalyst. The average CO_2_ conversions of the Ni/MEPCM and Ni/α-Al_2_O_3_ were almost 45% and 27%, respectively. In contrast, the value of the pure MEPCM was nearly 7%.

Since the pure MEPCM showed little catalytic activity, it was demonstrated that the Ni particles on the MEPCM drive catalytic activity. Moreover, the CO_2_ conversion of the Ni/MEPCM exceeded that of the Ni/α-Al_2_O_3_ by approximately 18%, suggesting that the MEPCM shell functions as a better catalyst support than ordinary α-Al_2_O_3_. This is attributed to the increase in the specific surface area of the MEPCM compared to that of the Ni/α-Al_2_O_3_. Whereas the Ni loaded onto each sample showed the ability to promote CO_2_ hydration, catalytic performances of Ni/MEPCM and Ni/α-Al_2_O_3_ were poor because the theoretical equilibrium of CO_2_ conversion was 97.50%, assuming only CH_4_ and CO were generated at 400 °C^36^.

**Figure 9:**
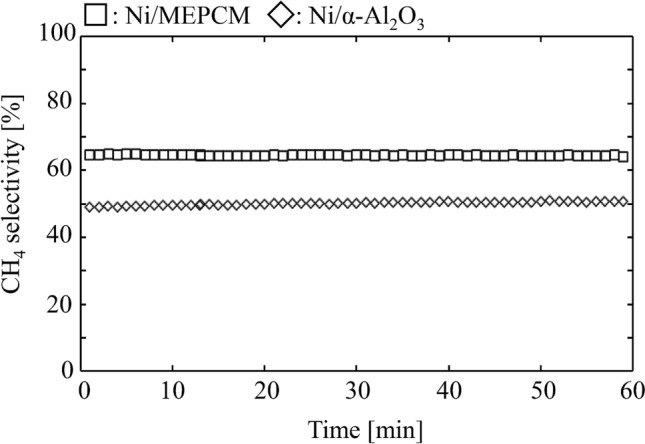
CH_4_ selectivity of the Ni/MEPCM and Ni/α-Al_2_O_3_. Reaction condition: ${F}_{in, {CO}_{2}}$= 10 ml min^−1^, ${F}_{in, {H}_{2}}$= 40 ml min^−1^, ${F}_{in, Ar}$= 110 ml min^−1^, Reaction temperature: 400 °C.

Figure 9 shows the CH_4_ selectivity of each catalyst. The average CH_4_ selectivity of the Ni/MEPCM and Ni/α-Al_2_O_3_ were almost 64% and 50%, respectively. Meanwhile, the value of the pure MEPCM could not be calculated because of the small amount of generated byproduct. Since the Ni/MEPCM possessed a higher CH_4_ selectivity than Ni/α-Al_2_O_3_, it is clear that the MEPCM can replace the α-Al_2_O_3_ support. The measurement errors were less than 2% for CO_2_ conversion and less than 0.5% for CH_4_ selectivity. Therefore, the measurements were precise and did not change the order of catalytic activity of each sample. The increase in the number of reaction sites was suspected as a factor for improving the CH_4_ selectivity. This could be inferred from the following three points: (1) the CO_2_ conversion of the Ni/MEPCM exceeded that of the Ni/α-Al_2_O_3_; (2) the surface area of the Ni/MEPCM was larger than that of the Ni/α-Al_2_O_3_; (3) SEM images showed aggregation of the NiO particles on the α-Al_2_O_3_.

CO_2_ methanation on Ni generally passes through CO as an intermediate^37–39^; therefore, a certain extent of CO production may be caused by incomplete reactions. It is assumed that the CO generation decreased due to the completion of the reaction as a result of the increase in the number of reaction sites. In the future, a detailed investigation of the surface properties of the Ni/MEPCM is needed to support this discussion. Additionally, both CH_4_ selectivities were lower than the theoretical equilibrium value, which is 99.87%, assuming only CH_4_ and CO were generated at 400 °C^36^. In this study, the amount of loaded Ni in the sample was 10 mass% of the total weight. When the amount of loaded Ni increases, the catalytic activity is expected to improve due to the increase in the number of reaction sites. However, the Ni/MEPCM faces a limitation in the form of a decrease in the heat absorption rate during thermal control. This is so because the decrease in heat storage density per mass depends on an increase in the amount of loaded Ni.

### Thermal control performance

**Figure 10:**
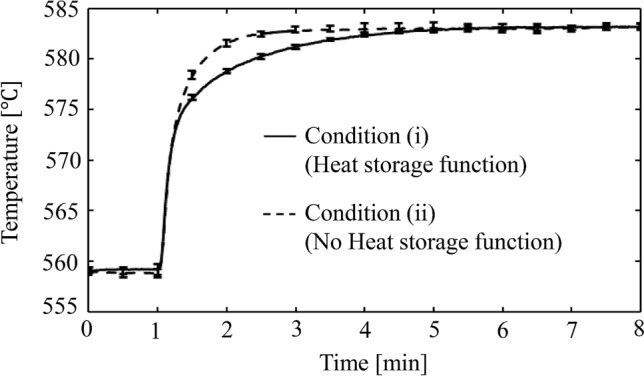
The temperature change of the Ni/MEPCM layer during CO_2_ methanation. Feed gas was introduced at 1 min after the starts of measurement. In the condition (i), the MEPCM has heat storage capacity. In the condition (ii), the MEPCM does not have it. Reaction condition: ${F}_{in, {CO}_{2}}$= 15 ml min^−1^, ${F}_{in, {H}_{2}}$= 60 ml min^−1^, ${F}_{in, Ar}$= 50 ml min^−1^, Reaction temperature: 560 °C. Each curve represents the average temperature of five measurements, and error bars indicate SD of temperature at each time.

Figure 10 shows the temperature change of the sample layer when CO_2_ methanation was performed under two conditions: with and without a heat storage capacity. These curves illustrate the average of five measurements, where feed gas was introduced 1 min after the measurement commenced. In both conditions, the temperature rose immediately after the injection of the feed gas. Without a heat storage capacity, the temperature of the sample layer increased linearly and stabilized at approximately 583 °C. When the sample had a heat storage capacity, the rising temperature trend behaved differently. The temperature of the sample layer rose linearly to approximately 573 °C, then gradually increased, stabilizing at approximately 583 °C. The error in each measurement was small, and the tendency of temperature rise was similar for all measurements under conditions (i) and (ii). Fig. S1 shows the concentration of the outlet gas obtained under conditions (i) and (ii). In both the conditions, the concentrations of CH_4_ and CO in the outlet gas were equal.

We considered that the final temperature stabilization was caused by the balance between the generated reaction heat and the heat lost to the outside of the reactor. A gradual temperature rise was observed only in the samples with a heat storage capacity. In addition, the gradual temperature rise started around 573 °C, which is close to eutectic temperature of the Al–Si alloy and the peak temperature of the phase transformation confirmed by DSC. Thus, we determined that the rate of increase of the temperature of the sample layer was lower because the thermal energy was expended on the phase change of the PCM (i.e., latent heat storage). It was confirmed that the catalyst-loaded MEPCM exhibited thermal control ability by slowing the rate of increase of the temperature of the catalyst layer. However, conditions (i) and (ii) exhibited similar catalytic activities. In general, the catalytic activities depend on the temperature of the catalyst. However, the temperature differences measured in this study were small, thus curbing definite differences in catalytic activity. Large-scale equipment is required to produce a definite temperature difference; however, this depends on whether the catalyst has a thermal storage capacity, and the difference in catalytic activity can be examined. Furthermore, the heat absorption rate of the Ni/MEPCM was calculated by Eq. (3).

3 $$W=\frac{{Q}_{0.2}}{\Delta t\times V}$$

In this formula, *W* (W m^−3^) represents the heat absorption rate, ${\text{Q}}_{0.2}$ (J) and *V* (m^3^) are the thermal storage capacity and volume, respectively, of 0.2 g of Ni/MEPCM, and $\Delta t$ (s) is the time during which the Ni/MEPCM stored heat. In this study, the loaded sample volume was approximately 59 $\times$ 10^–9^ m^3^. The theoretical heat storage capacity of the loaded sample was approximately 177.84 J, as determined by DSC. Therefore, ${\text{Q}}_{0.2}$ was 35.6 J. Since the temperature difference between the two curves clearly occurred between 1 and 5 min, the PCM completed the phase transformation in 4 min ($\Delta t=240 \mathrm{s}$). Therefore, the catalyst-loaded MEPCM exhibited an average rate of heat absorption of nearly 2.5 MW m^−3^. In this calculation, the contribution of the sensible heat storage was neglected as the temperature difference between the two curves was insignificant. The resulting heat absorption rate is competitive with the heat exchange rate of some chemical heat storage materials and heat transfer technology that utilize LHS^40–42^. This promising result comes from the fact that the MEPCM is a high-density energy storage device, and the novel structure of direct catalyst loading onto the PCM reduces the heat transfer distance.

## Conclusion

The use of MEPCM as a catalyst support was explored to control the local temperature rise of catalyst layers. MEPCM was fabricated from Al-Si microspheres. Ni was introduced as a catalyst for the CO_2_ methanation reaction by the impregnation method. The phase composition, morphology, and thermal storage capacity of the impregnated samples were evaluated. The catalyst performance of the Ni/MEPCM sample was investigated by estimating the CO_2_ conversion and CH_4_ selectivity when the CO_2_ methanation reaction was performed at 400 °C. In addition, the thermal control characteristics of the MEPCM during an exothermic reaction were investigated. The primary results were as follows:

1. The phase composition, morphology, and heat storage capacity of the pure MEPCM did not change even after processing via the impregnation method. Nano-sized NiO particles were dispersed on the surface of the Ni/MEPCM. Therefore, the impregnation method, which is a conventional method for supporting catalysts, is applicable to MEPCM, and smooth scale-up can be expected for industrial applications.
2. The Ni/MEPCM exhibited appropriate performance as a catalyst support, with a significant increase in CO_2_ conversion and CH_4_ selectivity compared to those of α-Al_2_O_3_ particles.
3. Because of the heat storage capacity of Ni/MEPCM, it absorbed the reaction heat at a favorable rate (2.5 MW m^−3^) and reduced the rate of increase of the catalyst layer temperature during the exothermic reaction. The risk of thermal runaway may be reduced by combining the material with existing cooling techniques and optimizing the feed gas conditions.

In conclusion, MEPCM can be used as an alternative catalyst support due to the excellent stability and activity of its ceramic shell. In the future, the optimization of MEPCM as a catalyst support using surface modification will be pursued to realize higher efficiency in material conversion to improve the economical aspect of the technique.

## Supplementary Information

Supplementary Information
