# Supplementary material for: Catalyst-loaded micro-encapsulated phase change material for thermal control of exothermic reaction
Source: Sci Rep. 2021 Apr 6;11:7539. doi: 10.1038/s41598-021-86117-1 (PMC8024387; doi:10.1038/s41598-021-86117-1)
Supplement: Supplementary file 1 — Supplementary Information [file 41598_2021_86117_MOESM1_ESM.pptx]

## Slide 1
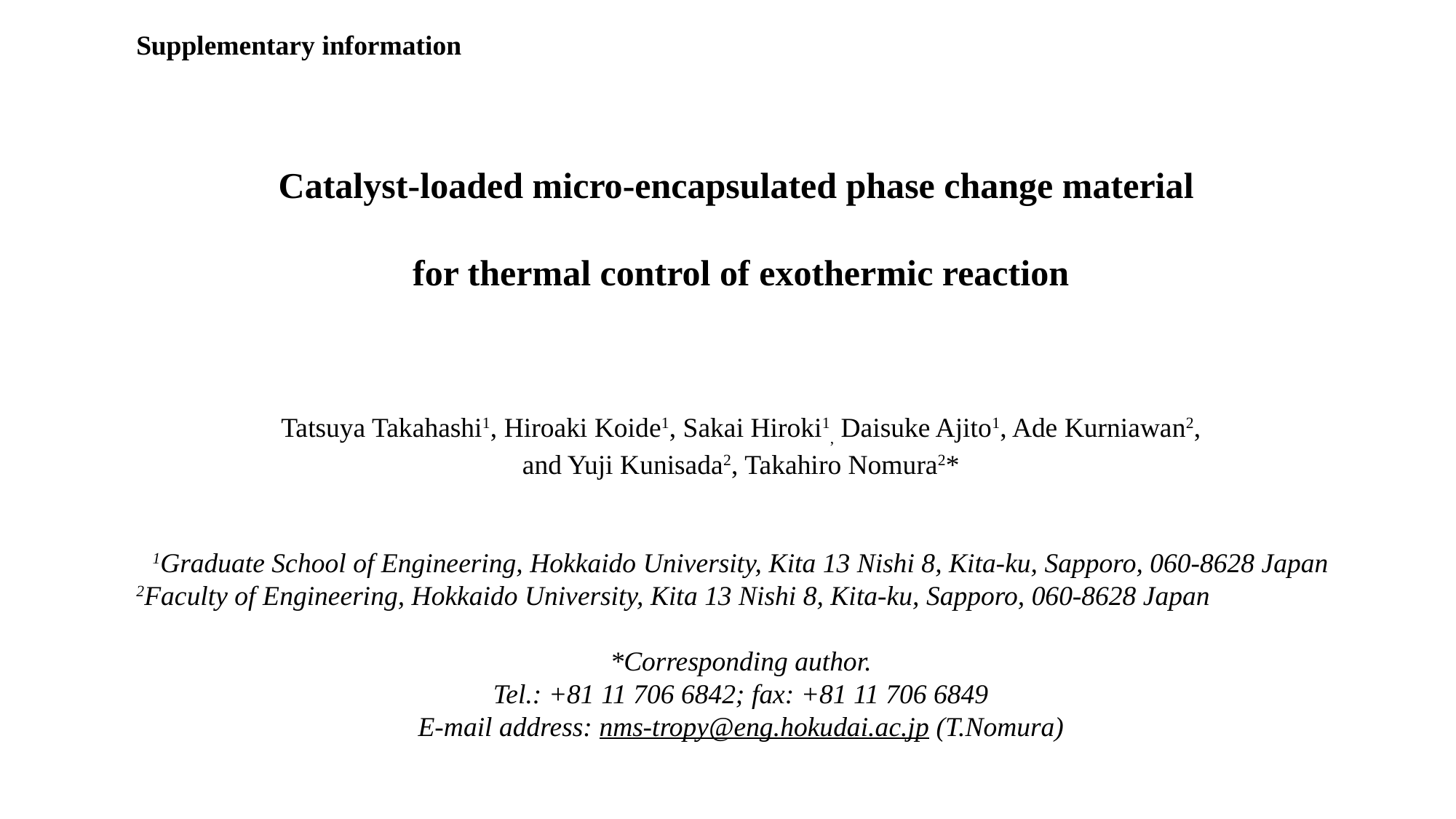

Supplementary information
Catalyst-loaded micro-encapsulated phase change material
for thermal control of exothermic reaction
Tatsuya Takahashi1, Hiroaki Koide1, Sakai Hiroki1, Daisuke Ajito1, Ade Kurniawan2,
and Yuji Kunisada2, Takahiro Nomura2*
1Graduate School of Engineering, Hokkaido University, Kita 13 Nishi 8, Kita-ku, Sapporo, 060-8628 Japan
2Faculty of Engineering, Hokkaido University, Kita 13 Nishi 8, Kita-ku, Sapporo, 060-8628 Japan
*Corresponding author.
Tel.: +81 11 706 6842; fax: +81 11 706 6849
E-mail address: nms-tropy@eng.hokudai.ac.jp (T.Nomura)

## Slide 2
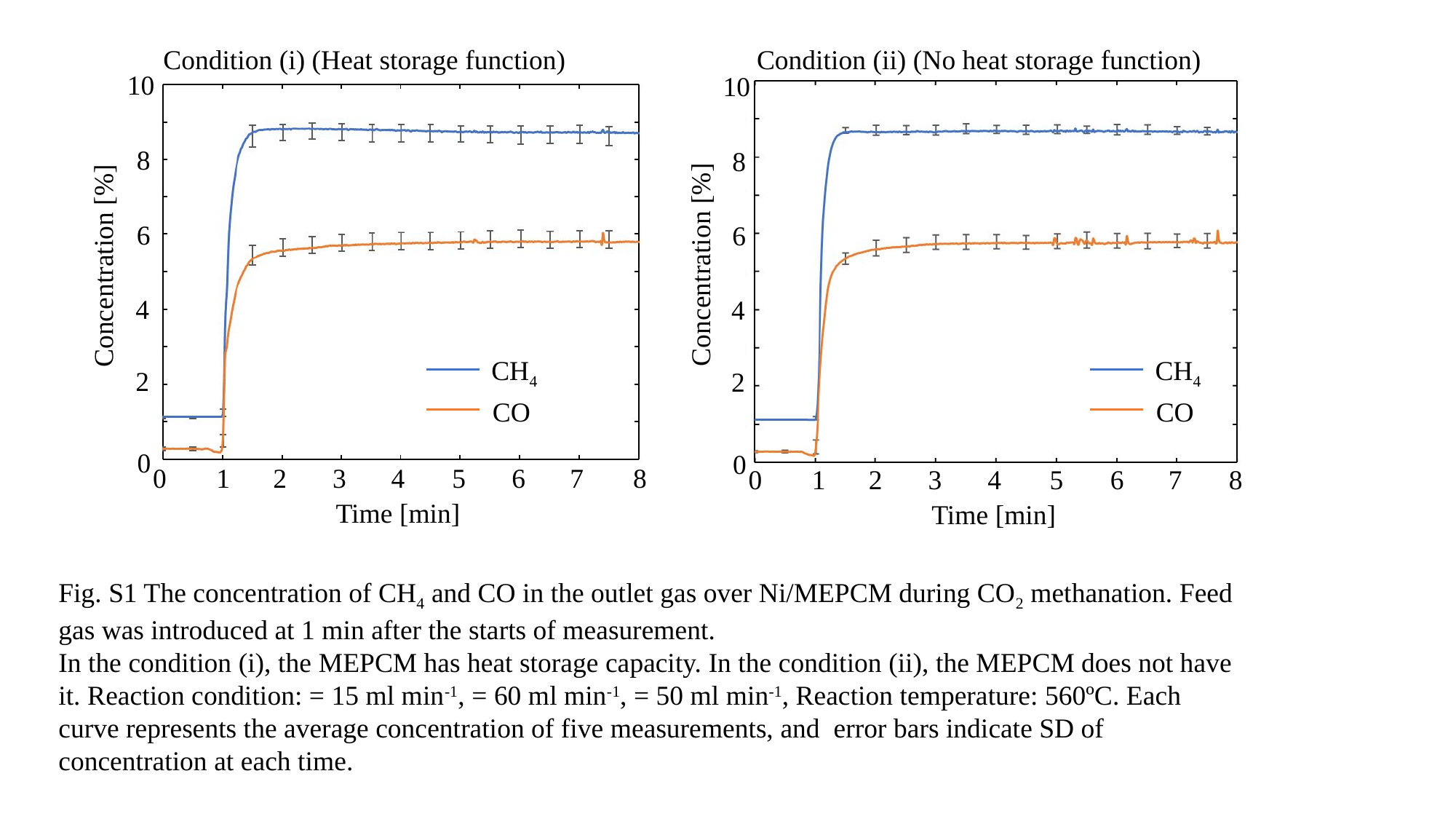

Condition (i) (Heat storage function)
Condition (ii) (No heat storage function)
10
10
8
8
6
6
Concentration [%]
Concentration [%]
4
4
CH4
CH4
2
2
CO
CO
0
0
0
1
2
3
4
5
6
7
8
0
1
2
3
4
5
6
7
8
Time [min]
Time [min]
